# The Role of Macrophages in Neuroinflammatory and Neurodegenerative Pathways of Alzheimer’s Disease, Amyotrophic Lateral Sclerosis, and Multiple Sclerosis: Pathogenetic Cellular Effectors and Potential Therapeutic Targets

**DOI:** 10.3390/ijms19030831

**Published:** 2018-03-13

**Authors:** Santa Mammana, Paolo Fagone, Eugenio Cavalli, Maria Sofia Basile, Maria Cristina Petralia, Ferdinando Nicoletti, Placido Bramanti, Emanuela Mazzon

**Affiliations:** 1 Department of Biomedical and Biotechnological Sciences, University of Catania, 95123 Catania, Italy; santa84ma@libero.it (S.M.); paolofagone@yahoo.it (P.F.); eugeniocavalli9@hotmail.it (E.C.); sofiabasile@hotmail.it (M.S.B.); m.cristinapetralia@gmail.com (M.C.P.); ferdinic@unict.it (F.N.); 2 IRCCS Centro Neurolesi “Bonino-Pulejo”, 98124 Messina, Italy; bramanti.dino@gmail.com; 3 Department of Formative Processes, University of Catania, 95124 Catania, Italy

**Keywords:** microglia, macrophages, neuroinflammation, Alzheimer’s disease, multiple sclerosis, Amyotrophic Lateral Sclerosis

## Abstract

In physiological conditions, different types of macrophages can be found within the central nervous system (CNS), i.e., microglia, meningeal macrophages, and perivascular (blood-brain barrier) and choroid plexus (blood-cerebrospinal fluid barrier) macrophages. Microglia and tissue-resident macrophages, as well as blood-borne monocytes, have different origins, as the former derive from yolk sac erythromyeloid precursors and the latter from the fetal liver or bone marrow. Accordingly, specific phenotypic patterns characterize each population. These cells function to maintain homeostasis and are directly involved in the development and resolution of neuroinflammatory processes. Also, following inflammation, circulating monocytes can be recruited and enter the CNS, therefore contributing to brain pathology. These cell populations have now been identified as key players in CNS pathology, including autoimmune diseases, such as multiple sclerosis, and degenerative diseases, such as Amyotrophic Lateral Sclerosis and Alzheimer’s disease. Here, we review the evidence on the involvement of CNS macrophages in neuroinflammation and the advantages, pitfalls, and translational opportunities of pharmacological interventions targeting these heterogeneous cellular populations for the treatment of brain diseases.

## 1. Introduction

**Figure 1:**
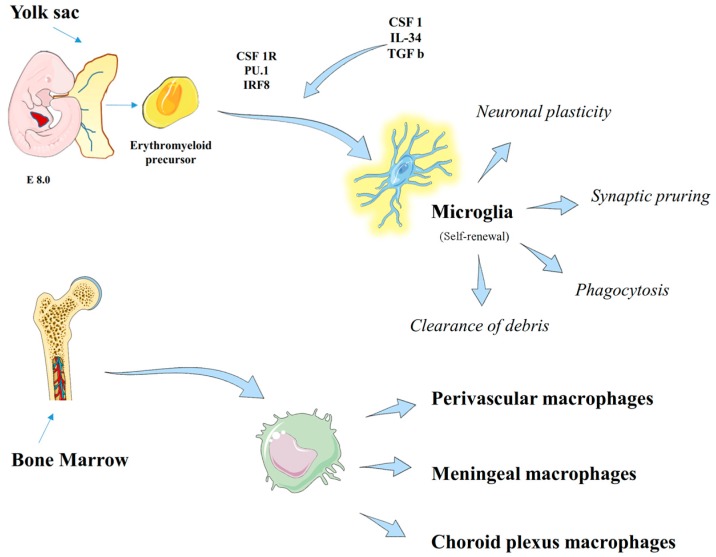
Origin of parenchymal and non-parenchymal CNS macrophages. This figure was drawn using the vector image bank of Servier Medical Art (http://smart.servier.com/). Servier Medical Art by Servier is licensed under a Creative Commons Attribution 3.0 Unported License (https://creativecommons.org/licenses/by/3.0/).

The central nervous system (CNS), as an “immuno-privileged” organ, hosts numerous populations of myeloid cells and defensive barriers such as the meninges, the perivascular space, and the choroid plexus [1]. Under steady-state conditions, the central myeloid cell populations in the CNS are represented by parenchymal microglia and non-parenchymal macrophages, namely perivascular macrophages, meningeal macrophages, macrophages of the choroid plexus, and blood-borne monocytes [2,3] (Figure 1). All of these populations are characterized by specific localization and molecular profiles [2,3]. Microglia are a unique tissue-resident macrophage population of the CNS and are considered to be primarily involved in immune reactions and inflammatory diseases [4]. 

Neuroinflammation involves a coordinated response between microglia and other CNS cells, such as astrocytes, as well as peripheral immune cells infiltrating the CNS. Several types of stimuli, including toxins, infections, trauma, or ischemia, elicit a rapid activation of the immune system, referred to as acute neuroinflammation, characterized by microgliosis and by the release of inflammatory mediators [5]. 

When this process is not regulated, it leads to chronic neuroinflammation, which, in turn, results in neurodegeneration, underlying several neurological disorders, including Alzheimer’s disease (AD), Amyotrophic Lateral Sclerosis (ALS), and multiple sclerosis (MS).

The characterization of microglia and brain macrophages and of their functions appears to be of primary importance to advance our knowledge of their role in the disease and may open up new therapeutic approaches.

## 2. Macrophage Populations in the Central Nervous System

### 2.1. Microglia Physiology

Microglia constitute from 5% to 12% of the total number of glial cells in the adult murine CNS [6] and, in humans, represents from 0.5% to 16.6% of glia with a higher density in the white than in the gray matter [7]. 

Recent fate-mapping studies of several macrophage populations in the body have provided elegant evidence that, under homeostatic conditions, microglia derive from mesodermal hematopoietic cells (HSCs) that originate in mammals from the yolk sac [8,9]. In vivo lineage tracing studies have shown that adult microglia derive from primitive myeloid progenitors arising before embryonic day 8 and that they are highly proliferative throughout early life [8].

Uncommitted c-kit^+^ stem cells that have both erythroid and myeloid potential are the direct yolk sac-derived precursors of microglia during early embryogenesis [10]. Microglia differentiation is independent from Myb, Inhibitor DNA-binding 2 protein HLH (Id2), Basic leucine zipper transcriptional factor ATF-like 3 (Batf3), and Kruppel-like factor 4 (Klf4) but dependent on the PU.1 and Interferon Regulatory Factor 8 (IRF8) pathways, and their survival is mediated by Colony Stimulating Factor 1 receptor (CSF1R) signaling [10,11]. The *PU.1* gene controls hematopoietic cell differentiation, as it is a vital target gene downstream of Runt-related transcription factor 1 (RUNX1) during embryonic hematopoiesis [12]. 

Mice with the *PU.1* gene knocked out were born alive but died of severe septicemia within 48 h [13] due to multiple hematopoietic aberrations, since they lacked mature B cells, circulating monocytes, and tissue macrophages, including microglial cells [13]. 

**Figure 2:**
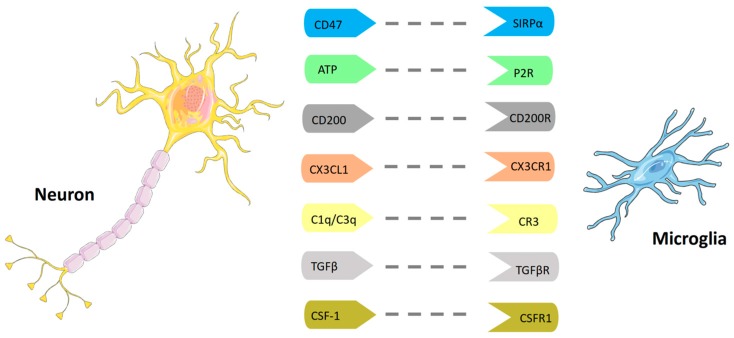
Cross-talk between microglia and neurons. This figure was drawn using the vector image bank of Servier Medical Art (http://smart.servier.com/). Servier Medical Art by Servier is licensed under a Creative Commons Attribution 3.0 Unported License (https://creativecommons.org/licenses/by/3.0/).

Neurons and microglia communicate with each other through the neuronal expression of several genes, including *Chemokine* (*C-X-C motif*) *ligand 1* (*CXCL1*), *CSF-1*, *Interleukin 34* (*IL-34*), and *Transforming Growth Factor beta 1* (*TGF-β1*). All microglial cells express CX3C chemokine receptor 1 (CX3CR1) in the brain and healthy neurons constitutively express high levels of chemokine (C-X3-C motif) ligand 1 (CX3CL1) (also named fractalkine). Decreased CX3CR1 expression negatively affects neurogenesis, leads to disruption of hippocampal circuit integrity, and impairs spatial learning and other behavioral and learning tasks [14] (Figure 2).

In mice, the differentiation of most macrophage populations is regulated by CSF-1 and the receptor CSF-1R. CSF-1R is expressed at a similar level in both yolk sac macrophages and microglia at embryonic day 9.5, and is maintained throughout development. Knockout of CSF-1R is associated with a reduced development of microglia and yolk sac macrophages but does not affect the number of circulating monocytes. IL-34 is a second ligand for CSF-1R, expressed in the brain at higher levels than CSF-1 [8]. In particular, IL-34 is detected prevalently in the neurons of the cortex, the anterior olfactory nucleus, and the hippocampus, but no expression is found in the brain stem and cerebellum [15]. Accordingly, in adult IL-34^LacZ/LacZ^ mice, microglia are partially reduced, predominantly in the brain areas with higher IL-34 expression [15]. On the other hand, in IL-34^LacZ/LacZ^ embryos, microglia precursors are present in a physiological number throughout development, suggesting that IL-34 is only required to maintain microglia homeostasis in specific regions of the adult CNS but not during embryonic development [15]. 

Also, reduced microglia are found in mice deficient in TGF-β1 in the CNS (CNS-TGF-β1^−/−^), therefore suggesting a role for TGF-β1 as a major differentiation factor. These mice, despite showing an apparently healthy behavior phenotype, have defects in glutamate recycling and synaptic plasticity and develop late-onset motor dysfunction in adult life [16]. 

Microglia show a unique transcriptomic signature, expressing transmembrane protein 119 (TMEM119), sialic acid-binding immunoglobulin-like lectin H (Siglec-H), P2Y purinoceptor 12 (P2RY12), Sal-like protein 1 (SALL1), CSF1R, TGFβ1, and TGFβ receptor 1 (TGFβR1). These molecules distinguish microglia from the non-parenchymal macrophages of the CNS [16,17,18,19]. 

In healthy CNS, microglia show a typical ramified morphology and express a variety of surface markers, including CD11b, CD45, EMR1 (also known as F4/80), the receptor CX3CR1 [14], CD200R1, CD172a/SIRPa, and TREM2b [20]. On the other hand, perivascular macrophages and meningeal macrophages have high expression of the mannose receptor, CD206. Finally, CNS macrophages can be distinguished from circulating monocytes by the expression of the Lymphocyte Antigen 6 Complex, Ly6C [11].

Microglia represent the primary immune effector cells of the cerebral parenchyma. Microglia contribute to the development of the brain and to its homeostasis, given their involvement in the programmed death of neuronal cells during development [21], the removal of cellular debris, dying cells or poorly folded, and in the regulation of neuronal synaptic plasticity [22] (Figure 1). 

In the healthy adult brain, microglial cells are highly dynamic in the resting state. The microglial cell somata are generally fixed, while microglial processes are active, with highly motile filopodia-like protrusions of different shapes. It is believed that the brain parenchyma undergoes complete screening by resting microglia once every few hours. This high resting motility may serve to perform housekeeping functions, enabling microglial cells to control the microenvironment efficiently and clear the parenchyma of metabolic end-products and damaged tissue. In the healthy brain, microglia interact with other cortical elements, i.e., astrocytes, neuronal cells, and blood vessels. However, when processes of nearby microglial cells overlap with each other, they are mutually withdrawn [23].

All brain macrophages, except for choroid plexus macrophages, are maintained locally throughout adulthood by self-renewal, rather than recruitment of circulating precursors to the CNS [24,25].

### 2.2. Microglia in CNS Pathology

An increasing body of data has demonstrated that microglia exert both neuroprotective and neurotoxic effects. Indeed, microglia produce inflammatory cytokines, such as Interleukin 1 beta (IL-1β), Tumor Necrosis Factor alpha (TNFα), Interleukin 6(IL-6), superoxide, nitric oxide, and excitatory amino acid [26], as well as neuroprotective factors such as neurotrophins, Brain-derived Neurotrophic Factor (BDNF), Glial cell-Derived Neurotrophic Factor (GDNF), and Nerve Growth Factor (NGF) [27].

In steady-state conditions, microglia express low levels of major histocompatibility class I and II complexes (MHC) molecules. However, once activated, microglia and CNS-infiltrating monocyte-derived macrophages upregulate many surface molecules, including major histocompatibility class II (MCHII) complexes and co-stimulatory molecules, which make them capable of presenting antigens to T cells more efficiently than astrocytes but less efficiently than dendritic cells [28]. Furthermore, under neuroinflammatory conditions, microglia express and release chemokines such as CCL2, CCL3, CCL4, CCL5, CXCL10, and CCL12, required for myeloid and T cell chemoattraction [29]. The surface molecule CD200 is widely expressed by neurons, astrocytes, and oligodendrocytes [30]. Its receptor CD200R (also named OX2 receptor) is primarily expressed on macrophages of the CNS, including microglia. The CD200–CD200R signaling leads to inactivation of microglia cells and keeps them in a resting state [31,32]. Indeed, microglia in *CD200^−/−^* mice form aggregates, associated with high expression of CD11b and CD45, particularly in the spinal cord [31]. Aggregation of microglia is usually observed during neuroinflammation and/or neurodegeneration [31]. In an animal model of multiple sclerosis (MS), the Myelin Oligodendrocyte Glycoprotein (MOG)-induced Experimental Allergic Encephalomyelitis (EAE) model, *CD200^−/−^* mice showed a more rapid onset of disease as compared to C57BL/6 *WT* mice. Accordingly, macrophage and microglia activation in the CNS of *CD200^−/−^* mice was dramatically enhanced, as determined by CD68 expression [31]. 

Following considerable CNS lesions, with extensive neuronal death, demyelination, or hemorrhage, damage-associated molecular patterns (DAMPs) are released and promote the morphological transformation of microglia in amoeboids, leading to significant alterations in gene expression. Nimmerjahn et al. have shown that laser-induced injury is associated with an early microglia response, characterized by movement of the nearby microglial processes toward the lesion. Only microglial cells adjacent to the lesion are found to be activated, and the number of activated cells is dependent on the severity of the injury [23]. 

Blood-derived monocytes express the chemokine receptors, C-C chemokine receptor 2 (CCR2) and CX3CR1. In rodents, CCR2 is absent from CNS myeloid cells and helps to distinguish infiltrating monocytes from resident myeloid cells. The constitutive expression of CCR2 on resident microglia is low if present. However, LPS and other pathological stimuli dramatically induce the expression of this receptor on microglia [33,34,35,36]. CCR2 and its ligands have been found to be upregulated in many types of CNS injury, including ischemia, hemorrhage, trauma, and hypoxia [37,38,39]. 

Chemokines and the nucleotide ATP, control the targeted migration of microglia in the damaged area. Many CNS cells, including neurons and astrocytes, release ATP as a transmitter, to allow intercellular communication [40]. Microglia activation, cytokine release, and phagocytosis are controlled by purinergic receptors. Low ATP concentrations represent a chemotactic stimulus for the recruitment of cells, while high levels of ATP also activate other effector functions, such as phagocytosis and cytokine secretion [41]. Microglia expresses multiple purinergic P2X and P2Y receptors, which regulate chemotaxis and phagocytosis [42]. P2X receptors are ligand-gated ion channel receptors, which comprise seven subtypes, all of which are primarily activated by ATP. All P2X receptor channels are permeable to Na^+^, K^+^, and Ca^2+^, while some of them are permeable to Cl^−^. P2Y receptors are seven-membrane-spanning, G-protein-coupled receptors, comprising eight subtypes, which are activated by ATP, ADP, UTP, and UDP [43]. 

In AD, evidence suggests that multiple microglia receptors, including CD36, CD47, integrinα6β1, Toll-like receptor (TLR) 4, TLR2, and scavenger receptor A, are co-activated in response to β-amyloid (Aβ). This causes the formation of a large receptor complex to mediate microglial phagocytosis of Aβ fibrils and the subsequent activation of pro-inflammatory intracellular signaling pathways [44,45,46]. 

FcγRs, by binding the Fc (fragment crystallizable) region of IgG immunoglobulin, are involved in a series of microglia functions, such as phagocytosis, oxidative burst, and cytokine release [47]. FcγR activation has been shown to stimulate inhibitory signaling through another microglial receptor, the signal regulatory protein α (SIRPα), which in turn inhibits FcγR- and complement receptor-mediated phagocytosis [48,49]. SIRPα, together with its ligand CD47, is also expressed by neurons and is involved in the regulation of neuronal apoptosis, neurite outgrowth, and synaptic activities [48,50,51]. In MS CD47 localizes in normal myelin but also in foamy macrophages and activated astrocytes surrounding the active MS lesions. The expression of CD47 has been found to be downregulated in MS brain lesions [52]. 

The Receptor for Advanced Glycation End-products (RAGE) exists in either a membrane-bound form or an insoluble form that lacks the transmembrane domain [53]. RAGE is highly expressed in the CNS cells including microglia, neurons, and endothelial cells and contributes to many pathological states characterized by an inflammatory component [54]. HMGB1-RAGE signaling links neuronal necrosis with microglia/macrophage activation. Therefore, RAGE signaling seems a potential target for anti-inflammatory therapy in stroke [55], as well as in other neuroinflammatory disorders.

Similar to peripheral macrophages, the classic versus alternative activated polarization state (also M1 and M2) paradigm has been proposed for microglia. M1 cells are usually associated with an acute infection, while M2 cells play a role in tissue remodeling, repair, and healing. The T helper 1 (Th1) cytokine, interferon-γ (IFNγ) [56], and bacterial lipopolysaccharide (LPS) polarize macrophages towards the M1 state and induce the release or expression of interleukin-(IL-)1, IL-6, IL-12, IL-23 and inducible Nitric Oxide Synthase (iNOS). In contrast, the presence of the Th2 cytokines, IL-4, IL-10, and IL-13 [56,57,58,59], transform microglia into M2 cells, which, in turn, produce IL-10 and express arginase 1. The chemokine CCL2, which is strongly induced in neurodegenerative and neuroinflammatory conditions, also drives M2 macrophage polarization [60,61]. M2 was further subdivided to accommodate similarities and differences in the effects provided by IL-4 (M2a), immune complex+ TLR ligands (M2b), IL-10, and glucocorticoid (M2c) stimuli [62]. Despite the usefulness of this categorization, however, Martinez and Gordon have highlighted the limitations of this schema, providing a viewpoint that undermines the possibility of applying the M1/M2 framework to microglia. To date, limited information is available on the microglial phenotypes that arise in neurological disorders, such as AD, ALS, and MS [63].

### 2.3. Non-Parenchymal CNS Macrophages

Non parenchymal CNS macrophages include perivascular, meningeal macrophages, and choroid plexus macrophages (Figure 1). All of them are CX3CR1^+^ CD11b^+^ CD45 high cells. All non-parenchymal macrophages were originally believed to originate from short-living blood monocytes after birth that is quickly replaced by bone marrow (BM)-derived cells. More recently, Goldmann and collaborators (2016) found that all of them have their prenatal origin in the yolk sac and depend on similar transcription factors (i.e., PU1., Irf8, Myb, and Batf3) [11]. Mice knocked out for *PU.1* do not have perivascular, meningeal, and choroid plexus macrophages. In *Irf8* knockout mice, a reduction of meningeal macrophages was observed, while the absence of Myb did not impair meningeal and choroid plexus macrophages. Finally, Batf3 deficiency did not affect any macrophage population. Also, Goldmann observed that while meningeal and perivascular macrophages are stable populations, choroid plexus macrophages undergo continuous exchange with peripheral blood cells [11].

Differently from microglia, perivascular macrophages express the mannose receptor, CD206, as detected in mice and humans [64], and CD163 in rats and humans [65]. Similarly, a significant proportion of meningeal macrophages express these receptors. Also, as compared to microglia, the perivascular and meningeal macrophages express higher levels of MHC antigens and show increased phagocytic activity [66]. Perivascular macrophages play a protective role during bacterial infection by recruitment of circulating leukocytes [67]. In a model of pneumococcal meningitides, depletion of the meningeal and perivascular macrophages was associated with a more aggressive disease course, characterized by higher blood and cerebrospinal fluid bacteria counts. Moreover, despite the presence of high levels of chemotactic factors (e.g., macrophage-inflammatory protein-2 and VCAM-1), a reduced number of white blood cells was observed in the cerebrospinal fluid.

Furthermore, it has been shown that they are involved in the preservation of endothelial cells integrity, the promotion of capillary stability, vascular constriction regulation, and the maintenance of BBB integrity [68].

## 3. Central Nervous System Macrophages in the Pathogenesis of Neuroinflammatory/Neurodegenerative Diseases

### 3.1. Alzheimer’s Disease

First described by Alois Alzheimer in 1907, Alzheimer’s disease (AD) is now the most common cause of dementia. Neuropathological hallmarks of the AD brain are Aβ accumulation, neurofibrillary tangles, synaptic loss, and neurodegeneration [69].

Mutations at or near the cleavage sites of β- and γ-secretase [70,71] or the mutations of γ-secretase constituents, *Presenilin-1* (*Psen1*) and *Presenilin-2* (*Psen2*) [72], result in increased production of Aβ and consequently lead to early onset of AD [73]. The polymorphism of *Apolipoprotein E* (*ApoE*) gene is a genetic risk factor for AD, with the ApoE4 allele strongly associated with an increased risk of AD, and the ApoE2 allele associated with protection [74].

The abnormal processing of amyloid precursor protein (APP) causes accumulation of insoluble Aβ, which induces free-radical reactions and inflammation and finally leads to the death of neurons and development of dementia [75,76,77].

Numerous epidemiological data seem to confirm the critical role of neuroinflammation in AD. Indeed, patients undergoing chronic treatment with non-steroidal anti-inflammatory drugs (NSAIDs) show a low risk of developing the disease, revealing the preventive effect of anti-inflammatory drugs [78]. Inflammatory components involved in AD-associated neuroinflammation include brain cells, such as microglia and astrocytes, the complement system, as well as cytokines and chemokines [79]. Microglia and bone-marrow-derived mononuclear phagocytes accumulate around senile plaques in AD patients and animal models of the AD [80,81,82]. It is also known that in AD, high concentrations of Aβ(1–40) or Aβ(1–42) do not cause neuronal damage if microglia are not present [83]. 

Microglia respond to the Aβ peptides and promote their clearance through the release of cytotoxic factors, which, in turn, promote the phagocytosis of these peptides [84]. Therefore, if, on the one hand, phagocytosis of amyloid-β peptides could improve disease course, on the other hand, the release of proinflammatory mediators seems to promote the disease [81].

Recent studies revealed multiple genetic risk factors for susceptibility to AD, including the polymorphic variants of the myeloid cell molecules, CD33 [85] and TREM2 [86]. CD33 is a cell surface molecule of the immunoglobulin superfamily that binds to sialic acids. A decrease in Aβ clearance by myeloid cells could be associated with the enhanced expression of CD33, which is a risk factor for late-onset AD [87,88]. TREM2 is transmembrane glycoprotein expressed in myeloid cells that transmit intracellular signals through its transmembrane binding partner DNAX-activating protein 12 (DAP12) [89]. Mutation in TREM2 decreased phagocytic capacities of microglia and is associated with higher Aβ load [90]. Also, in mouse models of AD, TREM2 deficiency reduces microglia recruitment around Aβ plaques, promoting their accumulation. Indeed, TREM2 represents a protective factor since it enables microglia to affect Aβ plaque depositions, therefore limiting damage of the neurons [91].

The signaling pathway CR3/C3 has also recently been implicated in early synapse loss in mouse models of AD-like pathology, suggesting that inappropriate activation of microglia by pathogenic proteins results in aberrant phagocytosis of functional synapses [92].

In addition, Khoury et al. have shown that the deletion of CCR2 in an AD mouse model resulted in a substantial reduction of microglial accumulation around the plaque and an increase in the deposition of Aβ [93], thus demonstrating that the chemokine CCR2 mediates the recruitment of inflammatory peripherals monocytes in the Alzheimer’s brain [94].

In vitro and in vivo studies show that the loss of neuron-microglial signaling CX3CL1/CX3CR1 leads to reduced Aβ deposition in two mouse models of AD, which is potentially mediated by altered activation and phagocytic capability of CX3CR1-deficient microglia [95]. 

The role of purinergic receptors has also been investigated. The role of P2X7 is controversial, and some studies show that the expression of P2X7 is increased in microglia in mouse models of AD or when microglia are stimulated with Aβ, suggesting its crucial role for microglial Aβ uptake [96,97]. Another study reported that silencing of P2X7 in microglia increased their capacity to clear Aβ, thus decreasing the rate of IL-1β release from microglia [98]. More evidence exists for the essential role of the P2Y2R in recruiting microglia to the brain during the development of AD. In a mouse model of AD, expression of CD11b, a marker for activated microglia, is elevated in hippocampal brain sections but reduced when the P2Y2R expression is suppressed. Also, the heterozygous deletion of the P2Y2R is associated with an increase in soluble and total Aβ1–42 levels and Aβ plaque deposition, a decrease in expression CD11b, and enhanced neurological deficits and accelerated mortality as compared to wild-type mice [99].

In the TgCRND8 mouse model of AD, depletion of perivascular macrophages significantly increased the vascular Aβ levels. Conversely, stimulation of perivascular macrophage turnover reduced cerebral amyloid angiopathy load, independently of clearance by microglia, highlighting the importance of the perivascular macrophages in brain disease [100]. 

### 3.2. Amyotrophic Lateral Sclerosis 

ALS is a neurodegenerative disease that belongs to the clinical and pathological spectrum of motor neuron disorders [101]. The disease is characterized by moderate and progressive dysfunction and loss of motor neurons. Neuronal injury depends upon well-orchestrated cross-talk between motor neurons and microglia [102]. ALS pathogenesis is often associated with aggregates of pathological superoxide dismutase 1 (SOD1), FUS (Fused in Sarcoma), or TDP-43 (TAR DNA-binding protein 43) in motor neurons and oligodendrocytes [103,104]. Neuroinflammation is a pathological hallmark of ALS and is characterized by the activation and proliferation of microglia and the infiltration of T cells into the brain and spinal cord [105].

Many studies indicate that microglial activation occurs before or concomitantly with the onset of clinical symptoms and increases during the disease course. Recent studies have shown that the in vivo activation state of microglia in ALS is characterized as a continuum between the neuroprotective M2 (alternatively-activated) phenotypic state and the neurotoxic M1 (classically-activated) state. In microglia from mutant *SOD1* (*mSOD1*) mice, at the onset of disease, higher levels of the M2 markers, Ym1, CD163, and BDNFand lower levels of the M1 marker, Nox2, can be observed as compared with end-stage disease. In addition, on the contrary to end-stage *mSOD1* M1 microglia, *mSOD1* M2 microglia in vitro resulted to be neuroprotective [106]. 

Beers and colleagues have evaluated in vivo the effects of microglia in the development of ALS by using *PU.1* knockout (*PU.1^−/−^*) mice, which at birth lack macrophages, neutrophils, T- and B cells, and microglia, and require bone marrow transplantation for survival. Transplantation of *mSOD1G93A* bone marrow into *PU.1^−/−^* mice did not show clinical or pathological evidence of motor neuron disease, indicating that mSOD1 in microglia alone is not sufficient to initiate disease [107].

Recruitment of inflammatory Ly6Chi monocytes to the spinal cord also has a pathological role in ALS. Treatment with anti-Ly6C mAb modulated the Ly6Chi monocyte cytokine profile, reduced monocyte recruitment to the spinal cord, diminished neuronal loss, and extended lifespan in a mouse model of ALS [108].

Both in ALS patients and in animal models, the hyperactivation of P2X7 receptors has been described in the advanced stages of the disease [109]. Indeed, the administration of P2X7 antagonist Brilliant Blue G (BBG) is able to delay onset and improve the general conditions and motor performance in SOD1-G93A mice, although without increasing lifespan [110]. 

### 3.3. Multiple Sclerosis

An increasing body of data suggests that CNS macrophages are involved in many neurological diseases, including MS. Studies have identified CCR2, CX3CR1, and the purine receptors P2X7 and P2X4 as crucial molecules involved in the etiopathogenesis of this disease.

MS is a demyelinating autoimmune disease of the CNS characterized by progressive axonal damage as a result of the loss of oligodendrocytes and neurodegeneration. 

Following blood–brain barrier damage, peripheral immune cells such as T lymphocytes, monocytes, and dendritic cells (DC) invade the CNS and co-activate the innate immune system within the CNS. T helper lymphocytes, mainly Th1 and Th17, cytotoxic T cells, B cells, macrophages, microglia, and the cytokines they secrete, are implicated in the initiation and maintenance of a deregulated immune response to myelin antigens and subsequent immune-mediated demyelination [111].

Microglia activation is regarded as a primary feature of neuroinflammatory diseases. However, in MS and experimental autoimmune encephalomyelitis (EAE), microglia have been shown to exhibit neuroinflammatory as well as neuroprotective characteristics. The activation of microglia precedes a massive immune cell infiltration and the demyelination process and finally dominates the remyelination and repair of disease [112]. In early active lesions, high levels of the phagocytic marker, CD68, as well as of MHC class I and II, and CD86 molecules are expressed by microglia and macrophages. On the other hand, in later stages of active lesions, an upregulation of the M2 activation markers CD206 and CD163 can be observed [113].

During the inflammatory process, various proinflammatory cytokines are produced, such as tumor necrosis factor (TNF) α, interferon (IFN) γ, IL-1β, IL6, and inducible iNOS, which can activate the microglial cells; these in turn increase the production of various proinflammatory factors, with consequent exacerbation of the symptoms of the disease.

Activated microglia are also the primary source of IFNβ in the inflamed CNS, which is thought to lead to increased phagocytosis of myelin debris at the peak of EAE, and treatment of naïve microglia with IFNβ improved removal of myelin debris in demyelinated organotypic cultures [114]. Genetic ablation of IFNβ or its receptor leads to increased severity of EAE [115]. 

Lampron and colleagues have found in mice with CX3CR1 deficiency that microglia clearance of myelin debris was significantly blocked, compromising the integrity of the myelin sheaths and thus preventing remyelination [116]. Given the pro-inflammatory role of the purinergic receptor P2X7, its involvement in the etiopathogenesis of MS has been investigated. Sharp and collaborators have observed that in P2X7-deficient mice, the incidence of EAE disease was reduced compared to the wild-type mice [117]. Also, treatment with P2X7 antagonists of chronic EAE reduces demyelination and ameliorates the associated neurological symptoms [118]. 

Another target for the treatment of multiple sclerosis is CCR2, which is expressed in inflammatory monocytes. CCR2 and its corresponding ligand, CCL2, are associated with numerous neuroinflammatory conditions. CCL2 is synthesized in the CNS, mainly by astrocytes, and to a lesser extent by microglia, endothelial cells, and neurons [119] and controls the infiltration of inflammatory monocytes into the inflamed brain [120]. Accordingly, lack of CCR2, or the deletion or inhibition of CCL2, reduces monocyte-derived macrophage recruitment into the CNS in mice with EAE [121] and is associated with less severe EAE disease scores [122,123].

Finally, another study showed that conditional deletion of transforming growth factor (TGF)-β-activated kinase 1 (TAK1) in CX3CR1+ tissue macrophages, suppressed CNS inflammation, and decreased axonal damage by cell-autonomous inhibition of the NF-κB, JNK, and ERK1/2 pathways in EAE [124].

### 3.4. Myeloid-Targeted Therapy

Targeting the CNS myeloid cell populations represents a promising therapeutic avenue for many CNS disorders. Many targets have been identified, including High Mobility Group Box 1 (HMGB1), Adenosine Monophosphate-activated Protein Kinase (AMPK), Peroxisome Proliferator-Activated Receptor Gamma (PPARγ), and Glycogen Synthase Kinase 3 beta (GSK3β), and several drugs are now being tested for their potential neuroprotective profiles. 

HMGB1 is a non-histonic chromosomal protein that acts as a proinflammatory cytokine since damaged neurons release it and it is secreted from activated macrophages. A massive release of HMGB1 has been observed in primary cortical cultures upon NMDA- or glutamate-induced excitotoxicity [125]. Primary microglia cultures incubated with media from NMDA-treated primary cortical cells underwent significant activation, as determined by NO secretion and expression of the proinflammatory factors, TNF-α, cyclooxygenase-2 (COX2), and iNOS [125]. Accordingly, immunoneutralization of HMGB1 restored basal levels of NO production. Also, a supernatant from short hairpin (sh) *HMGB1*-expressing cortical cells was not sufficient to induce microglia activation [125]. In AD, HMGB1 is associated with senile plaques and seems to inhibit microglial Aβ42 clearance, thereby increasing Aβ42 neurotoxicity. By binding HMGB1 and HMGB2, the small molecule inflachromene blocks their post-translational modifications and release, and, in turn, reduces microglial activation [126,127,128]. It has also been reported that Glycyrrhizin, a triterpene extracted from the roots and rhizomes of the plant *Glycyrrhiza glabra* (licorice), binds directly to HMGB1, inhibiting its chemoattractant and mitogenic activities [125].

Activation of the AMP-activated protein kinase (AMPK) has been found to be associated in vitro with reduced NF-κB activation and a consequent decrease in the expression of pro-inflammatory cytokines and iNOS in glial cells. Several natural and synthetic molecules are known activators of AMPK, including berberine, resveratrol, metformin, and 5-amino-4-imidazole carboxamide riboside. In vitro and in vivo data have confirmed the ability of these molecules to exert neuroprotective effects in a variety of settings, including Aβ-induced neurotoxicity [129,130,131]. In an EAE model, phosphorylated and total levels of AMPK are reduced during onset and peak of disease, but increase in the remission phases. Moreover, modulation of AMPK signaling follows the expression of IFN-γ and the IFN-γ-induced chemokine CCL2 in the brain [132]. More recently, it was shown that the Angiotensin II type 1 receptor blocker telmisartan promoted M2 polarization and reduced M1 polarization in LPS-challenged in microglia cells via enhancing AMPK activation. Indeed, the effects of telmisartan were reduced by AMPK knockdown or administration of an AMPK inhibitor [133] and were prevented by treatment with a siRNA for Ca^2+^/calmodulin-dependent protein kinase kinase β (CaMKKβ), an upstream kinase of AMPK [133]. It should be noted that the neuroprotective effects of telmisartan are also partly dependent on its effects as an AT1 receptor blocker and PPARγ partial agonist [134], as the decrease of neuronal injury and microglia activation by telmisartan is the result of AT1 receptor blockade and PPARγ activation [135,136]. Along the same lines, the PPARγ activator pioglitazone reduced neuron damage and improved survival in the G93A-SOD1 transgenic mouse model of ALS, and reduced neuroinflammation in mouse models of AD, thus improving disease severity. However, clinical data are still disappointing, as pioglitazone in combination with riluzole has shown no effects on the survival of ALS patients [137,138,139].

Promising data also come from pharmacological targeting of the glycogen synthase kinase-3β (GSK3β). The phosphatidylinositol 3-kinase (PI3K)/Akt signaling pathway, as well as protein kinase C and protein kinase A, are major regulators of GSK3. In turn, GSK3 promotes inflammation, as its activity has been found necessary for the full induction of cytokine production, upon TLR stimulation (reviewed in [140]). Treatment of BV-2 microglia with GSK3 inhibitors (i.e., lithium, indirubin-3′-monoxime, and kenpaullone) significantly decreased the migration of cells and reduced the production of IL-6 and NO upon LPS stimulation [141]. Finally, GSK3β inhibitors have been showed to be neuroprotective in mouse models of ALS, delaying the onset of symptoms and prolonging animal lifespan [142,143]. 

The cannabinoid receptors represent another promising target as they may induce a shift from M1 to the M2 phenotype. In resting microglia, low or no expression of either CB1 or CB2 can be detected. CB2 receptors are upregulated in the activated microglia, as has been found in brain tissue from AD and MS patients (reviewed by [144]). In both acute and chronic models of EAE, the administration of an endocannabinoid receptor ligand, 2-arachydonyl-glycerol, delayed the onset of disease and ameliorated the disease course by increasing the number of M2 macrophages in the perivascular infiltrations [145]. Also, the phytocannabinoid cannabidiol (CBD) modulates microglial cell function in vitro and improves an in vivo model of AD [146].

Several classes of antipsychotic drugs, such as dopamine D2 receptor antagonists and selective serotonin reuptake inhibitor, have also been shown in vitro to reduce IFNγ-induced microglial activation and suppress the release of pro-inflammatory cytokines [147,148,149].

Interestingly, novel pharmacological delivery tools such as poly(methyl methacrylate) nanoparticles (PMMA-NPs) have been designed in order to be able to enter specifically into activated microglia/macrophages and release pharmacologically active compounds, such as pioglitazone, minocycline, and rolipram, which have been shown to modulate microglia activation in different preclinical models [150]. 

Finally, gene transfer vehicles able to target microglial cells have been tested in preclinical models to regulate cellular activation. To this aim, recombinant vectors based on adeno-associated virus (rAAV) as gene transfer vehicles have been designed. The rAAV can be used to transfer genes into mammalian cells, where it integrates specifically within short genomic regions. In order to generate microglia-specific AAV-derived vectors, cell-type-specific mammalian promoters can be used, such as the regulatory elements for human CD11b and CD68, as well as murine F4/80 [151].

A summary of myeloid-targeted therapies is presented as Table 1.

## 4. Conclusions

Many neurodegenerative and autoimmune CNS disorders are still orphan diseases, and in the long term the current therapeutic approaches are highly ineffective. It is now believed that targeting the CNS myeloid populations may represent a promising strategy, aimed at modulating cellular activation and switching the cellular phenotype from neurotoxic to neuroprotective. Indeed, anti-inflammatory approaches have failed to be effective, particularly in neurodegenerative diseases, both in animal models and in clinical trials. Many potential microglial targets have been identified, and several molecules are currently being tested in preclinical models. However, despite the encouraging results, much more effort is needed to progress these molecules into the clinical setting. Also, it is of primary relevance to continue expanding basic knowledge on microglia/CNS macrophages biology in order to identify key genes and signaling pathways that regulate CNS homeostasis and potentially control the pathophysiology of CNS diseases.

## Figures and Tables

**Table 1:** Myeloid-targeted therapy.

Treatment	Target	Disease	Effect	References
Inflachromene	HMGB1–HMGB2	Animal Model of AD	Blocks post-translational modifications and releases reduce microglial activation	[126,127]
Berberine Resveratrol	AMPK AMPK	LPS- and IFN γ BV-2 microglia cells Animal model of AD Animal model of AD	Reduces neuroinflammation Ameliorates neurotoxicity induced by Aβ. Improves the cognitive impairment	[129,130,131]
Telmisartan	AMPK PPARγ	LPS-challenged microglia cell	Promotes M2 polarization and reduces M1 polarization Reduction of neuronal injury and microglia activation	[133]
Pioglitazone	PPARγ	Animal model of ALS Animal models of AD	Reduces neuron damage and increases survival Reduces neuroinflammation, Improves disease severity	[137,138,150]
GSK3β inhibitors	GSK3β	LPS-challenged BV-2 microglia cell Animal model of ALS	Reduces IL-6 and NO Delays onset of symptoms and prolongs the lifespan	[141,142,143]
2-arachydonyl-glycerol Cannabidiol (CBD)	Endocannabinoid receptor	Experimental Allergic Encephalomyelitis Animal model of AD	Improves disease course Prevents the cognitive impairment	[145,146]
Risperidone, Perospirone and Quetiapine	D2 receptor	IFN γ activated microglia cells	Suppresses the release of pro-inflammatory cytokines	[147,148,149]

## Abbreviations

CNS	Central nervous system
AD	Alzheimer’s disease
ALS	Amyotrophic lateral sclerosis
MS	Multiple sclerosis
HSCs	hematopoietic cells
CSF1R	CSF1 receptor
IL-34	Interleukin-34
CNS-TGF-β1^−/−^	mice deficient in TGF-β1 in the CNS
TMEM119	transmembrane protein 119
Siglec-H	sialic acid-binding immunoglobulin-like lectin H
P2RY12	P2Y purinoceptor 12
SALL1	Sal-like protein 1
TGFβ1	transforming growth factor-β1
TGFβR1	TGFβ receptor 1
BDNF	Brain-derived neurotrophic factor
GDNF	Glial cell-derived neurotrophic factor
NGF	Nerve growth factor
MCHII	major histocompatibility class II
MOG	Myelin oligodendrocyte glycoprotein
EAE	Experimental allergic encephalomyelitis
DAMPs	damage-associated molecular patterns
CCR2	C-C chemokine receptor 2
Fc	fragment crystallizable
SIRPα	signal regulatory protein α
RAGE	Receptor for advanced glycation end-products
IL	Interleukin
iNOS	inducible nitric oxide synthase
Th1	T helper 1 (Th1)
IFNγ	interferon-γ
LPS	lipopolysaccharide
TLR	Toll-like receptor
Psen1	Presenilin-1
Psen2	Presenilin-2
ApoE	Apolipoprotein E
APP	amyloid precursor protein
NSAIDs	non-steroidal anti-inflammatory drugs
DAP12	DNAX-activating protein 12
SOD1	superoxide dismutase 1
FUS	Fused in Sarcoma
TDP-43	TAR DNA-binding protein 43
mSOD1	mutant SOD1
PU.1^−/−^	PU.1 knockout
BBG	Brilliant Blue G
DC	dendritic cells
ROS	reactive oxygen species
TNF	tumor necrosis factor
TGF	transforming growth factor
TAK1	transforming growth factor (TGF)-β-activated kinase 1
Sh	short hairpin
AMPK	AMP-activated protein kinase
CaMKKβ	calmodulin-dependent protein kinase kinase β
GSK3β	glycogen synthase kinase-3 β
PI3K	The phosphatidylinositol 3-kinase
CBD	cannabidiol
PMMA-NPs	poly(methyl methacrylate) nanoparticles
rAAV	recombinant vectors based on adeno-associated virus
